# Supplementary material for: MdGRF22, a 14-3-3 Family Gene in Apple, Negatively Regulates Drought Tolerance via Modulation of Antioxidant Activity and Interaction with MdSK
Source: Plants (Basel). 2025 Jun 27;14(13):1968. doi: 10.3390/plants14131968 (PMC12252163; doi:10.3390/plants14131968)
Supplement: Supplementary file 1 [file plants-14-01968-s001.zip › plants-3690547-supplementary.doc.pdf]

Table S1. Protein sequence of *MdGRF22* and homologous gene.

Gene	Protein sequence	Length (bp)
MdGRF22	MAASSPREENVYVAKLAEQAERYEEMVEFMEKVVSSLPEGE<br>EPTVEERNLLSVAYKNVIGARRASWRIVSSIEQKEESRGNTD<br>HVATIKEYRARIENELSNICGGILKVLESKLVPSAKVGESKVF<br>YLKMKGDYHRYLAEFKTGDERKEAAENTLNAYKAAQDIA<br>TSELAPTHPIRLGLALNFSVFYYEILNSPDRACSLAKQAFDEA<br>IAELDTLGEDSYKDSTLIMQLLRDNLTLWTSDMQDDGTDEI<br>KEAPKPQAEPQQ	261
XM_009347515.3	MAALSPREENVYVAKLAEQAERYEEMVEFMEKVVSSLPEGE<br>EPTVEERNLLSVAYKNVIGARRASWRIVSSIEQKEESRGNTD<br>HVATIKEYRARIENELSNICGGILKVLESKLVPSAKVGESKVF<br>YLKMKGDYHRYLAEFKTGDERKEAAENTLNAYKAAQDIA<br>TSELAPTHPIRLGLALNFSVFYYEILNSPDRACSLAKQAFDEA<br>IAELDTLGEDSYKDSTLIMQLLRDNLTLWTSDMQDDGTDEI<br>KEASKPQAEPQQ	261
XM_028199692.1	MATPTPREDNVYMAKLAEQAERYEEMVEFMEKVSAAVCD<br>SEELTVEERNLLSVAYKNVIGARRASWRIISSIEQKEESRGNA<br>DHVATIKDYRTKIESELSSICDGILKLLDSMLIPSATSGDSKVF<br>YLKMKGDYHRYLAEFKTGAERKEAAENTLNAYKAAQDIA<br>NTELAPTHPIRLGLALNFSVFYYEILNSPDRACSLAKQAFDE<br>AIAELDTLGEDSYKDSTLIMQLLRDNLTLWTSDMQDDGAEE<br>IKEAPKHEEEKQ	260
XM_016883872.2	VEERNLLSVAYKNVIGARRASWRIVSSIEQKEEGRGNADHV<br>AVIRDYRAKIEAELSEICAGILKLLDENLVPAAGNGDSKVFY<br>LKMKGDYHRYLAEFKTGDDRKSAAENTLTAYKSAQDIAVA<br>ELAPTHPIRLGLALNFSVFYYEILNSPDRACSLAKQAFDEAIA<br>ELDTLGEDSYKDSTLIMQLLRDNLTLWTSDMQDDGTDEIKE<br>ASKPEEEKQP	217
XM_021763282.1	MAAGSPREDNVYMAKLAEQAERYEEMVQFMEKVVFSTPES<br>DELTVEERNLLSVAYKNVIGARRASWRIISSIEQKEESRGNA<br>DHVAAIKEYRAKIEGELSEICGGILKLLDEKLVPAAASGDSK<br>VFYLKMKGDYHRYLAEFKTGNERKEAAENTLNAYKSAQDI<br>ANAELAPTHPIRLGLALNFSVFYYEILNSPDRACNLAKQAF<br>DEAISELDTLGEDSYKDSTLIMQLLRDNLTLWTSDMQDDGT<br>DEIKDAAKREEEQKQ	261
NM_001280923.1	MAAAPSAREENVYMAKLAEQAERYEEMVEFMEKVSAAVD<br>SEELTVEERNLLSVAYKNVIGARRASWRIISSIEQKEESRGND<br>HHVAMIRDYRSKIESELSSICDGILKLLDSRLIPSASSGDSKVF<br>YLKMKGDYHRYLAEFKTGAERKEAAESTLTAYKSAQDIAN<br>AELAPTHPIRLGLALNFSVFYYEILNSPDRACNLAKQAFDEA<br>IAELDTLGEESYKDSTLIMQLLRDNHTLWTSDMQDDGADEI<br>KEAPKRDDEQQ	260
XM_028084528.1	MASAPSPREEFVYMAKLAEQAERYEEMVEFMEKVSAAAEN	261

	EELTVEERNLLSVAYKNVIGARRASWRIISSIEQKEESRGNED<br>HVAVIRDYRSKIESELSNICDGILKLLDSRLVPSAASGDSKVF<br>YLKMKGDYHRYLAEFKTGADRKEAAESTLSAYKSAQDIAN<br>SELPPTHPIRLGLALNFSVFYYEILNSPDRACSLAKQAFDEAI<br>AELDTLGEESYKDSTLIMQLLRDNLTLWTSDMQDDGADEIK<br>EAAPKGDGEQN	
XM_024313861.2	MAAPTPREENVYMAKLAEQAERYEEMVEFVEKVSASADKE<br>ELTVEERNLLSVAYKNVIGARRASWRIISSIEQKEESRGNDD<br>HVSMIREYRSKIETELSKICDGILKLLDSRLIPSAFSGDSKVFYL<br>KMKGDYHRYLAEFKTGAERKEAAESTLTAYKAAQDIANAE<br>LAPTHPIRLGLALNFSVFYYEILNSPDRACNLAKQAFDEAIA<br>ELDTLGEESYKDSTLIMQLLRDNLTLWTSDMQDDGADEIKE<br>APKPTEEAKQ	260
XM_020381996.2	MASAPSPREEFVYMAKLAEQAERYEEMVEFMEKVSAAVEN<br>EELTVEERNLLSVAYKNVIGARRASWRIISSIEQKEESRGNED<br>HVAVIRDYRSKIEAELSNICDGILKLLDSRLVPSAAAGDSKVF<br>YLKMKGDYHRYLAEFKTGADRKEAAESTLSAYKSAQDIAN<br>TELPPTHPIRLGLALNFSVFYYEILNSPDRACSLAKQAFDEAI<br>AELDTLGEESYKDSTLIMQLLRDNLTLWTSDMQDDGADEIK<br>EAAPKGDGEQN	261

Table S2. Primers sequences.

Gene	Use	Sequences
MdGRF22	q-PCR	F: 5′-GAGGAGCCCACCGTCGAAGAG-3′<br>R: 5′-GGAGGAGACGATACGCCAGGAG-3′
pCAMBIA1300-MdGRF22	Gene clone	F: 5′-GAGCTCGGTACCCGGGGATCCATGGCGGCCTCATCCCCA-3′<br>R: 5′-CATGTCGACTCTAGAGGATCCCTGCTGTGGCTCAGCCTGA-3′
pGBKT7-MdGRF22	Gene clone	F: 5′-AGGCCGAATTCCCGGGGATCCTTATGGCGGCCTCATCCCCA-3′<br>R: 5′-CCGCTGCAGGTCGACGGATCCTCACTGCTGTGGCTCAGCCT-3′
pGADT7-MdSK	Gene clone	F: 5′-GTGGGCATCGATACGGGATCCTTATGGCTGACGATAAGGAAATTTCT-3′<br>R: 5′-CAGCTCGAGCTCGATGGATCCTTATGTGCCAGCCGGATGC-3′
AD universal	PCR	F: 5′-TAATACGACTCACTATAGGG-3′<br>R: 5′-AGATGGTGCACGATGCACAG-3′
Hyg	Identification	F: 5′-AGCTCGGTACCCGGGGATCCATGGCGGCAACCACCCCC-3′<br>R: 5′-GGTGTCGACTCTAGAGGATCCGGTCTCGATCTTGGATTGGTAGTC-3′
MdGAPDH	actin	F: 5′-GAGCTCGCAGGTATCCTTTCT-3′<br>R: 5′-TACCAAGCAATGACCTTGACC-3′

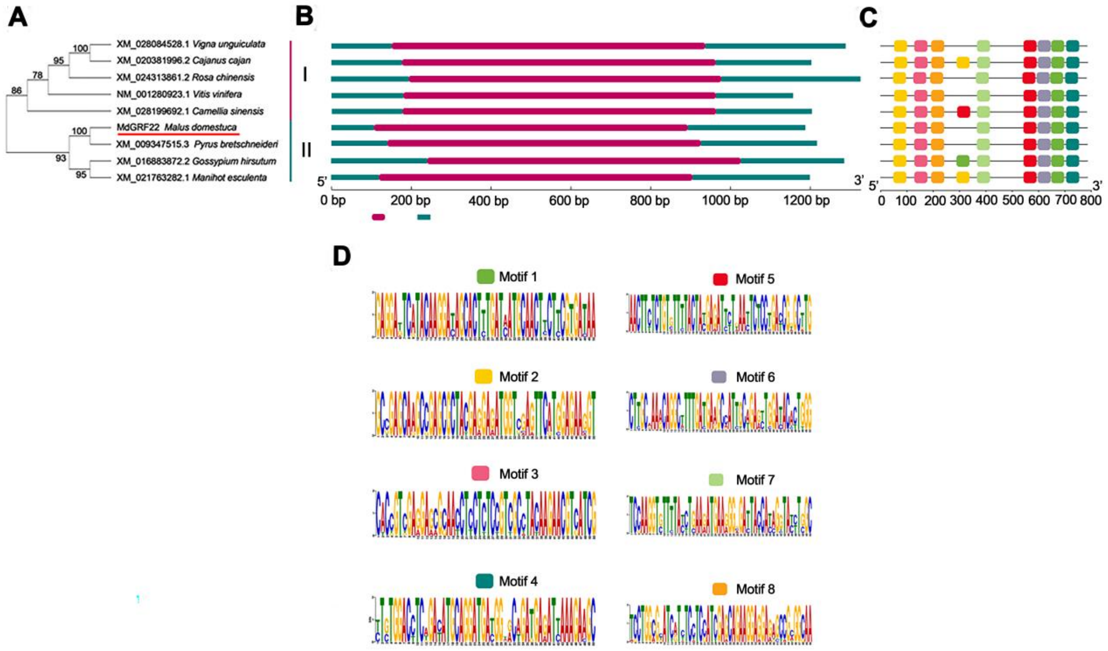

Figure S1. Sequence characterization and analysis of *MdGRF22* and homologous genes. **(A)** Phylogenetic tree of *MdGRF22* and homologous genes. The tree was constructed using the NJ method with 1000 bootstrap replicates. **(B)** Gene structure analysis of *MdGRF22* and homologous genes. **(C)** Motif analysis of *MdGRF22* and homologous genes. **(D)** A total of eight Motifs were identified.

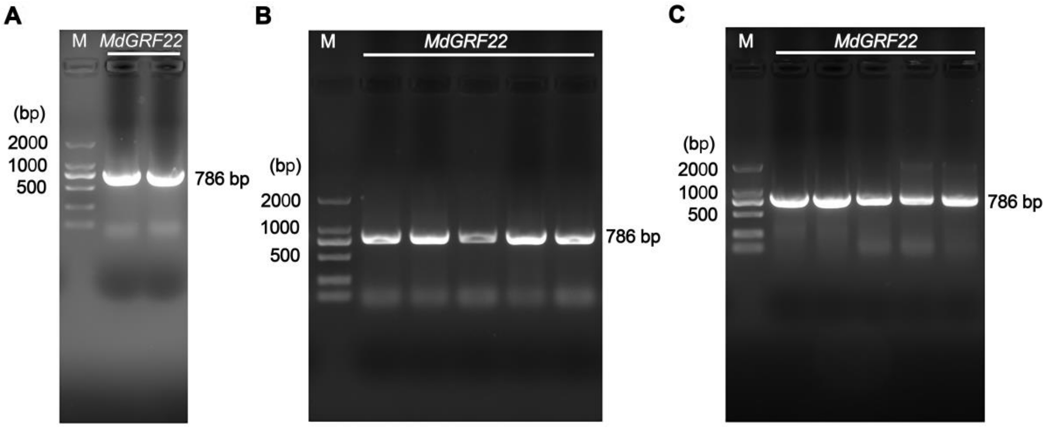

Figure S2. PCR amplification map of *MdGRF22*. **(A)** Gene cloning of *MdGRF22*. **(B)** *MdGRF22* gene transformation of E. coli. **(C)** *MdGRF22* gene transformation of agrobacterium. Of these, M represents the 2000 bp Marker, and the white horizontal line indicates PCR-positive products.

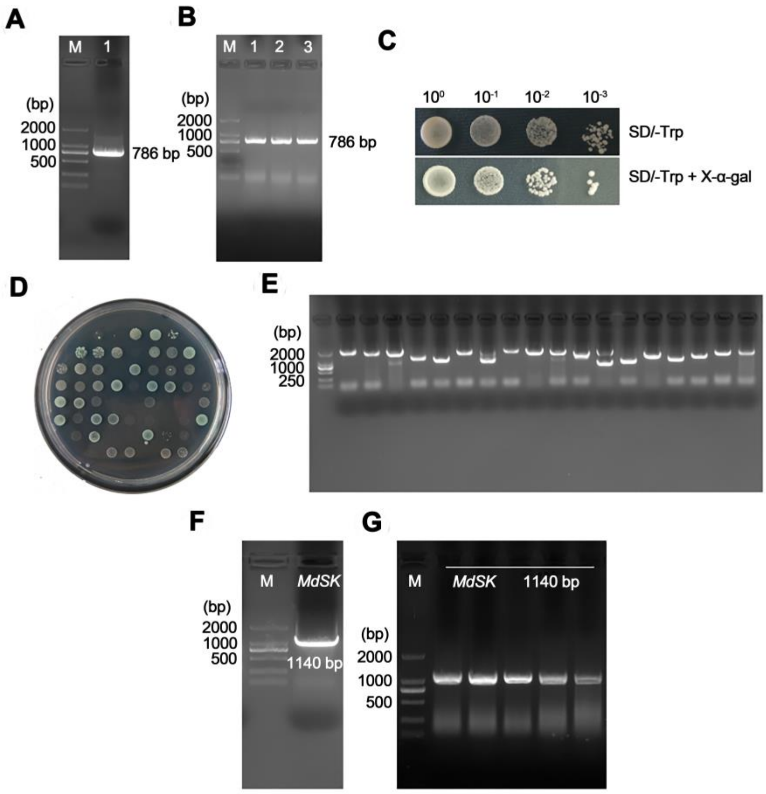

Figure S3. Analysis of the *MdGRF22* protein in the Y2H sieve library. **(A)** Gene cloning of *MdGRF22* and **(B)** E. coli transformation of *MdGRF22*, respectively. Here, M indicates the Marker, and lanes 1, 2 and 3 show positive amplification products. **(C)** Validation of the self-activation of the MdGRF22 protein. **(D)** Screening of the MdGRF22-pGBKT7 library identified several blue-stained strains and underwent PCR amplification. **(E)**, **(F)** and **(G)** are *MdSK* gene cloning and E. coli transformation, respectively.
